# Temperature Compensation of Laser Methane Sensor Based on a Large-Scale Dataset and the ISSA-BP Neural Network

**DOI:** 10.3390/s24020493

**Published:** 2024-01-12

**Authors:** Songfeng Yin, Xiang Zou, Yue Cheng, Yunlong Liu

**Affiliations:** 1 School of Electronics and Information Engineering, Anhui Jianzhu University, Hefei 230601, China; yinsongfeng@tsinghua-hf.edu.cn (S.Y.); 17730006008@163.com (Y.L.); 2 Hefei Institute for Public Security, Tsinghua University, Hefei 230601, China; chengyue@tsinghua-hf.edu.cn; 3 Hefei Tsingsensor Technology Co., Ltd., Hefei 230601, China

**Keywords:** laser methane sensors, temperature compensation, isolation forest algorithm, BP neural network, sparrow search algorithm

## Abstract

We aimed to improve the detection accuracy of laser methane sensors in expansive temperature application environments. In this paper, a large-scale dataset of the measured concentration of the sensor at different temperatures is established, and a temperature compensation model based on the ISSA-BP neural network is proposed. On the data side, a large-scale dataset of 15,810 sets of laser methane sensors with different temperatures and concentrations was established, and an Improved Isolation Forest algorithm was used to clean the large-scale data and remove the outliers in the dataset. On the modeling framework, a temperature compensation model based on the ISSA-BP neural network is proposed. The quasi-reflective learning, chameleon swarm algorithm, Lévy flight, and artificial rabbits optimization are utilized to improve the initialization of the sparrow population, explorer position, anti-predator position, and position of individual sparrows in each generation, respectively, to improve the global optimization seeking ability of the standard sparrow search algorithm. The ISSA-BP temperature compensation model far outperforms the four models, SVM, RF, BP, and PSO-BP, in model evaluation metrics such as MAE, MAPE, RMSE, and R-square for both the training and test sets. The results show that the algorithm in this paper can significantly improve the detection accuracy of the laser methane sensor under the wide temperature application environment.

## 1. Introduction

Natural gas is widely used as a clean energy source in various fields, but its main component, methane (CH_4_), is flammable and explosive [1,2]. Although methane gas is colorless, odorless, and non-toxic, its leakage can easily lead to severe combustion and explosion accidents, resulting in significant casualties and property damage [3,4]. Especially in environments such as coal mines, methane concentrations are often deficient but sufficient to form explosive mixtures [5]. In emergencies, methane concentrations can quickly reach even tens of percent. The problem with methane, however, is not only its combustion and explosion risks but also its greenhouse effect [6]. Since the Industrial Revolution, global warming has significantly threatened human society and ecosystems. Methane is not only a greenhouse gas; its greenhouse effect is 22 times greater than carbon dioxide, making it the second globally warming greenhouse gas in the earth’s atmosphere after carbon dioxide [7,8,9]. Therefore, how to detect methane gas concentration more effectively and accurately in complex application environments has become the target of attention and research direction for many researchers [10].

Laser methane sensors can be used for real-time online monitoring of natural gas leaks in complex environments. However, laser methane sensors are sensitive to temperature, resulting in a significant difference between the detected CH_4_ concentration and the actual value [11]. Commonly used temperature compensation methods include hardware compensation and software algorithm compensation. Since hardware compensation is easily affected by circuit components and welding accuracy, resulting in the lack of precision of gas concentration detection results, but also has the defect of high cost, most scientists use software compensation methods such as the least squares method and polynomial fitting method. It has the advantages of high programmability, lower price, and scalability [12,13,14]. Considering that the effect of temperature on the detection results of laser methane sensors includes a variety of factors such as gas molecules, optical components, and circuit components [15], there is still a significant error between the corrected concentration value and the actual value by the above method. Some scholars have improved the prediction effect of laser methane sensors and simplified the temperature compensation process by establishing a neural network temperature compensation model with higher prediction accuracy, strong generalization ability, and learning ability [16].

Machine learning algorithms have various applications in various technical aspects of gas sensors. Xiaonan Liu demonstrated in detail the application of utilizing shallow neural network (SNN) fitting algorithms for the spectral data processing domain to achieve denoising. This neural network has the advantages of simple structure and robustness [17]. In addition, among many machine-learning-based methods for temperature compensation, we cite the following that reflect the state-of-the-art:

SVM [18]: Methane gas temperature compensation using a Support Vector Machine (SVM) algorithm to train regression models. The sensor detects the temperature and corresponding concentration values as inputs, and the temperature-compensated predicted methane concentration is used as an output. However, SVM is sensitive to parameter tuning and unsuitable for training large data samples. This leads to poor temperature compensation when a large number of data samples are used for training.

Random Forest [19]: The structure of a random forest consists of multiple decision trees. In each decision tree, nodes are split by randomly selecting a subset of features and samples until a certain depth or purity is reached. Random forests can handle complex nonlinear relationships and are resistant to overfitting. However, random forest models are more complex and require higher memory and computational resources, which leads to longer training time on large datasets.

BP Neural Network [20]: The method takes advantage of the fact that BP neural networks can backpropagate the error to compensate for the nonlinear errors due to temperature variations. However, the BP neural network tends to fall into local optimization, and the probability of overfitting and underfitting increases.

PSO-BP Neural Network [21]: The primary purpose is to optimize the weights and biases of the BP neural network using the Particle Swarm Optimization (PSO) algorithm, which better enables the neural network to perform in predicting methane concentration. However, the PSO algorithm’s global optimization ability could be more stable under significant temperature variations, leading to insufficient prediction accuracy under extreme temperature conditions or when the temperature range changes too much.

The prediction accuracy for the temperature compensation model based on a neural network mainly depends on training samples and the network model structure. The aim of this paper is twofold. Firstly, to deal with the need for large-scale datasets training laser methane sensors, we created thousands of large-scale data by adding the data cleaning algorithm based on Improved Isolation Forest [22] to the actual laser methane sensor high and low-temperature methane concentration detection. Secondly, this paper introduces the Improved Sparrow Search Algorithm (ISSA) [23] to optimize the BP neural network for global optimization and better generalization ability. Temperature-compensated prediction through data cleaning and model training will lead to higher prediction accuracy and generalization for laser methane sensors. There are different types of Laser methane sensors. The most commonly used detectors are based on TDLAS technology, including Direct Absorption Spectroscopy (DAS) and Wavelength Modulated Spectroscopy (WMS). Photoacoustic Spectroscopy (PAS), although often used in conjunction with TDLAS, works differently from TDLAS in that it detects acoustic waves generated by gas molecules absorbing a modulated laser [24]. In this paper, we utilize the advantages of DAS in terms of simplicity of operation, cost-effectiveness, and stability to conduct a temperature compensation study of a laser methane sensor. However, the large-scale dataset cleaning and temperature compensation methods we designed and studied can also be applied to various fields and spectroscopic techniques.

## 2. Establishment of Large-Scale Dataset

### 2.1. Data Acquisition and Segmentation

**Figure 1:**
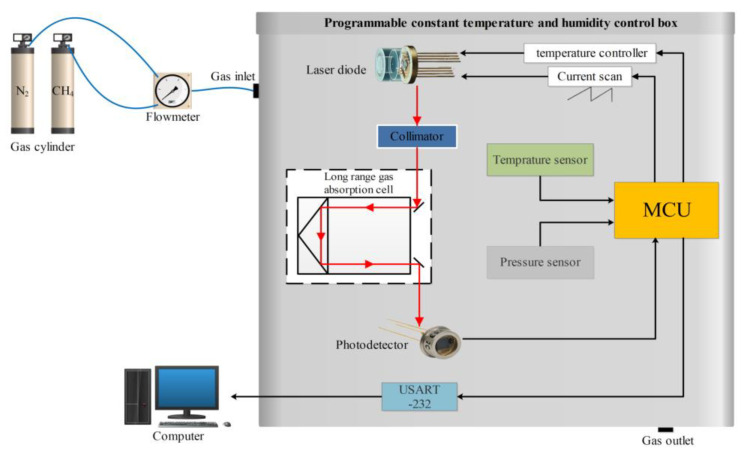
Experimental equipment for testing the effect of temperature on CH_4_ gas concentration.

The essential equipment employed under laboratory conditions to determine the influence of temperature on the CH_4_ gas concentration detected using TDLAS is illustrated in Figure 1. The laser methane detector mainly includes a DFB laser, an open, reflective gas chamber, a circuit board, a photodetector, a pressure sensor, and a temperature sensor. Among them, the laser has a center wavelength of 1653.72 nm, a drive current of 20–50 mA, and a drive voltage of 3.3 V. The open-reflective gas chamber includes two gold-plated mirrors. Mounting holes for the laser, photodetector, temperature, and pressure sensors are provided at the bottom. The top of the circuit board is mounted with an MCU master control chip, laser temperature control chip, temperature sensor, memory chip, resistor, and capacitor. The experimental system mainly consists of the gas distribution system, the light source emission part, the gas absorption cell, the data receiving and processing unit, and the programmable constant temperature and humidity box. The light source emission part mainly consists of a distributed feedback (DFB) laser and a semiconductor laser controller.

**Figure 2:**
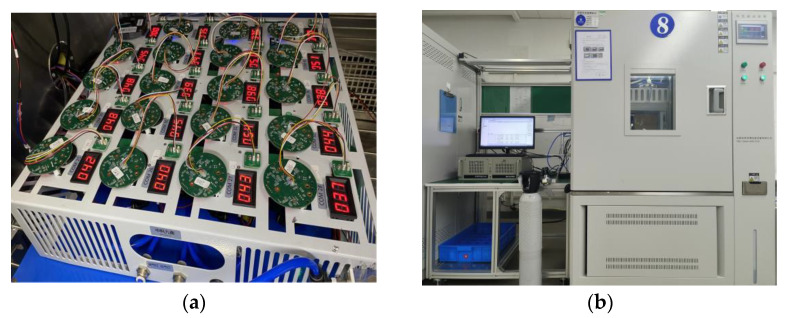
High and low-temperature experimental equipment of laser methane sensor: (**a**) Photo of laser methane sensor; (**b**) High and low-temperature calibration.

The data collected in this paper are the ambient temperature sensor detections and methane concentration values detected by the laser methane sensor in low-temperature (−20~0 °C), normal-temperature (10~30 °C), and high-temperature (40~65 °C) environments. Within each temperature interval, we slowly ramped the temperature from −20 °C, taking measurements in 5 °C steps and selecting stable laser methane sensor temperature and concentration values during the ramp-up process. Figure 2 shows the photographs of the equipment required for the laser methane sensor to collect data for high- and low-temperature experiments.

This paper collected 15,810 sets of sensor temperature and concentration data from laser methane sensors, thus creating large-scale datasets to serve as the base data for temperature compensation studies. The datasets include data obtained at low, normal, and high temperatures, with each temperature interval containing CH_4_ measurements at standard concentrations of 0.5%, 2%, and 8%. As shown in Table 1, the datasets were divided into training and test datasets to improve the prediction accuracy of the established temperature compensation model. To select the temperature compensation model. The training data set consists of 2800 records of 2% and 8.0% CH4 concentration detection data, corresponding to different ambient temperatures. The test datasets contain data from untrained temperatures (−10~0 °C, 10~15 °C, and 40~55 °C) from 0.5%, 2%, and 8% CH_4_ concentration data.

In order to ensure the completeness and randomness of the sample data used for model training, we obtained 15,810 sets of detection data from 9160 laser methane sensors in high- and low-temperature experiments at different temperatures and concentrations. We established a large-scale dataset to ensure its completeness and representativeness. In addition, data preprocessing cleaning is performed on the collected data to eliminate as much noise and outliers as possible in the dataset to ensure the quality of the large-scale measured data. Finally, to ensure the randomness of the data used for temperature compensation model training, we use random sampling to train the data, which reduces the potential selection bias and enhances the model’s generalization ability.

### 2.2. Data Preprocessing with Improved Isolation Forest Outlier Detection Algorithm

When the laser methane sensor collects high- and low-temperature test data and builds a large-scale dataset before temperature compensation, there may be some anomalous concentration data due to different sensor use times and hardware performance differences. The abnormal data can significantly impact the prediction effect of the temperature compensation model for gas concentration.

Isolation Forest (IForest) is a typical class of unsupervised anomaly detection algorithms in integrated learning algorithms. High- and low-temperature gas concentration detection can change significantly with temperature, and the IForest algorithm uses a random selection of features and segmentation points for the segmentation of the data, which can lead to inaccurate and meaningless segmentation and limitations in the accuracy of data cleaning. This paper proposes an Improved Isolation Forest (IIForest) algorithm. We add the K-Means++ clustering algorithm [25] to the basic IForest algorithm. The steps of the IIForest-based high- and low-temperature gas concentration detection data cleaning method are as follows:

Step 1: Data preparation. Experimentation and data sampling of laser methane sensors at different ambient temperature conditions.

Step 2: Construction of Isolated Forest. A set of decision trees is constructed using the traditional Isolated Forest algorithm.

Step 3: Automatic selection of the number of clusters. Firstly, the data is clustered using the K-Means++ algorithm to select an appropriate *K* value to divide the data into *K* clusters. Secondly, the cluster center is calculated as a representative point of the data subset for each cluster, and the clustering error is calculated. Finally, the location where the inflection point occurs in the 2D data consisting of the clustering error and the number of clustered clusters is taken as the optimal number.

Step 4: The K-Means++ algorithm is used in the traditional process of constructing the tree of an isolation forest to divide the dataset into several optimal clustering clusters, and each optimal clustering cluster is used as a branch of the isolated forest tree. During the construction of each decision tree, the dataset is divided into clusters rather than randomly selecting data division points. This allows for better differentiation between each cluster and provides more accurate anomaly scores. For each data point x, the membership grade is calculated based on its path length in the tree and the total path length of the tree.

Step 5: Data Cleaning. Anomaly detection is performed on the dataset using the IIForest method. The anomaly score is calculated based on each data point’s path length and cluster size in the decision tree. The formula for calculating the anomaly score *S(x, T)* is

(1) $$S(x,\;T)=2^{\left(-\frac{E(h(x))}{c(m)}\right)}$$

where *h*(*x*) is the path length of data point x in tree *T*, *E*(*h*(*x*)) denotes the expected value of the path length, and *c*(*m*) is a constant given depth *m* is a constant.

**Figure 3:**
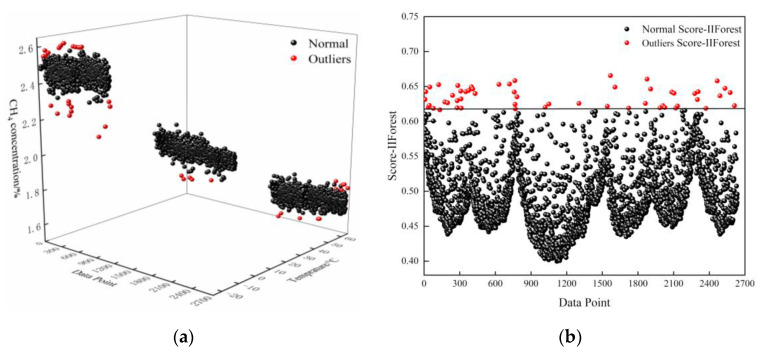
IIForest algorithm outlier detection results: (**a**) 2% CH_4_ gas concentration data clustering classification and outlier detection effect map; (**b**) IIForest algorithm rating scores for 2% CH_4_ gas concentration data.

Figure 3 shows a graph of the outlier detection results of the experimental CH_4_ data at 2% concentration using the IIForest algorithm. The distribution of data types after the outlier removal of the training set data by the IIForest algorithm is shown in Table 2.

## 3. ISSA-BP Temperature Compensation Methods

### 3.1. ISSA-BP Temperature Compensation Models

Due to the complex mechanisms by which the gas is to be measured, and the various components in the laser methane sensor are affected by temperature, it is not easy to ensure stable CH_4_ concentration output accuracy by a single polynomial fitting model [26,27,28]. The sensor temperature compensation algorithm based on a neural network model has good generalization and learning ability, and better compensation results can be obtained from a large number of training datasets [29]. The neural network training method and datasets have an essential influence on the prediction accuracy. In this paper, we propose the ISSA-BP model with global optimization capability to improve the prediction accuracy of temperature compensation.

Based on the temperature compensation model of ISSA-BP, we set the number of nodes in the input layer to 2, the number of nodes in the output layer to 1, the number of hidden layers to 1, and the loss function is defined as Mean Square Error (MSE). As shown in Table 3, the detection data of 2% standard concentration of CH_4_ gas in the temperature range of −20 °C to 65 °C were selected for the cyclic experiment. At the hidden layer node number of 5, the MSE is 3.23 × 10^−5^, and the optimal hidden layer node number can be determined compared to other node numbers.

**Figure 4:**
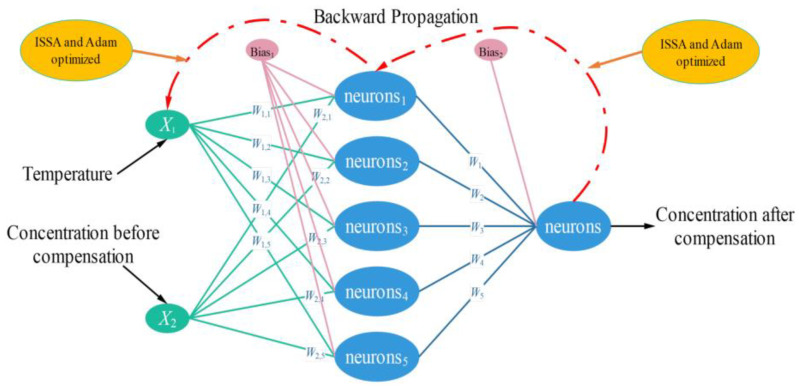
ISSA-BP Neural Network temperature compensation model structure.

As shown in Figure 4, the model structure of ISSA-BP Neural Network in the temperature compensation model, which takes Sensor temperature detection value and CH_4_ concentration before compensation as the data of the input layer of the model, and the output is the predicted value of the concentration after temperature compensation.

### 3.2. Improved Sparrow Search Algorithm

#### 3.2.1. Quasi-Reflective-Based Learning Strategies Initialize Populations

The standard sparrow search algorithm (SSA) initializes the population using a random function. This method results in a lack of diversity of sparrow populations and needs to improve on problems such as uneven distribution within the search space. This paper uses the Quasi-reflective-based Learning strategy [30] (QRBL), which can quickly perform a wide range of searches to initialize the population. Let a feasible solution for the current population in the j-dimensional search space be $X_{j}=(x_{1},x_{2},\ldots ,x_{j})$. Its quasi-reflective solution is $\bar{X_{j}}=(\bar{x_{1}},\bar{x_{2}},\ldots ,\bar{x_{j}})$, The position of the quasi-reflective solution is given by

(2) $$\bar{X_{j}}=rand(({lb}_{j}+{ub}_{j})/2,\;X_{j})$$

where *X_j_* is a sparrow individual in the j-dimensional search space; $X_{j}\in \left[{lb}_{j},{ub}_{j}\right]$; *lb* and *ub* are the lower and upper bounds in the algorithm parameters, and ∀j∈1,2,...,j.

#### 3.2.2. Explorer Location Update Strategy Improvements

Since the position update formula for the explorer position in the standard SSA algorithm when the warning value is less than the safety threshold is

(3) $$X_{i,j}^{t+1}=X_{i,j}^{t}\cdot \exp\left(-\frac{i}{\alpha \cdot {iter}_{max}}\right)$$

where $X_{i,j}^{t}$ is the *j*-dimensional value of the *i* sparrow at iteration number *t*; ${iter}_{max}$ is the maximum number of iterations. Since the search range of the explorer particles in this formula gradually decreases and tends to 0, it affects the convergence speed of the algorithm. It causes the algorithm to fall into the local optimum easily.

To solve the aforementioned problems, this paper introduces the randomized prey search strategy in the Chameleon Swarm Algorithm [31] (CSA) to improve the explorer position update. This position update strategy can improve the information exchangeability between populations and prevent them from falling into local optimality. The improved sparrow explorer position update formula is

(4) $$X_{i,j}^{t+1}=\left\{\begin{array}{c} X_{i,j}^{t}+\mu \left(\left({ub}_{j}-{lb}_{j}\right)r_{1}+{lb}_{j}\right)sgn\left(\mathrm{rand}-0.5\right),{\;R}_{2}<ST\\ X_{i,j}^{t}+Q\cdot L,{\;\;\;\;\;\;\;\;\;\;\;\;\;\;\;\;\;\;\;\;\;\;\;\;\;\;\;\;\;\;\;\;\;\;\;\;\;\;\;\;\;\;\;\;\;\;\;\;\;\;\;\;\;\;\;\;\;\;\;\;\;\;R}_{2}\geq ST \end{array}\right.$$

where *r*_1_ is a random number within (0, 1); *μ* is the convergence factor, calculated as: $\mu =\gamma {exp\left(-\frac{\alpha t}{{iter}_{max}}\right)}^{\beta }$, the values of *γ*, *α*, and *β* are taken as 1, 3.5 and 3; rand is a random number within (0, 1); *Q* is a normally distributed random number; *R*_2_ is a hazard warning value, *ST* is an indication of the safety threshold.

#### 3.2.3. Anti-Predator Location Update Strategy Improvements

The global optimal position of the antipredator in the standard SSA algorithm for the ith sparrow, the antipredator position update formula when it realizes the danger and escapes is

(5) $$X_{i,j}^{t+1}=X_{i,j}^{t}+K\cdot \left(\frac{\left|X_{i,j}^{t}-X_{worst}^{t}\right|}{\left(f_{i}-f_{w}\right)+\varepsilon }\right)$$

where *X*_worst_ is the current worst position in the world; *K* denotes a random number in [−1, 1]; *f_i_* is the current fitness value of the individual sparrow; *f_w_* is the current global worst fitness value; *ε* is a constant that avoids the denominator being zero, *ε* = 1 × 10^−10^.

The anti-predator individual in this formulation is at the current global optimum, so the search range of its particles is reduced, increasing the probability that the algorithm is premature. To solve the aforementioned problems, this paper introduces the Levy flight strategy [32] with a randomized step size, which can achieve a more extensive search area when searching in an unknown location, thus improving the global search capability of the anti-predator. The improved anti-predator position update formula is

(6) $$X_{i,j}^{t+1}=\left\{\begin{array}{c} X_{best}^{t}+\beta \cdot \left|X_{i,j}^{t}-X_{best}^{t}\right|,\;\;\;\;\;\;\;\;\;\;\;\;\;\;\;\;\;\;\;\;\;\;f_{i}>f_{g}\\ X_{i,j}^{t}+\alpha \cdot \left|X_{i,j}^{t}-X_{worst}^{t}\right|\cdot Levy(\xi ),\;\;\;{\;f}_{i}=f_{g} \end{array}\right.$$

where $X_{best}^{t}$ is the location of the current optimal solution; α denotes the randomized step size after repeated experiments to take the value of 0.55; *f_g_* denotes the current global best fitness value. For Levy(ξ) is usually represented by Mantegna’s algorithm [33], whose randomized search path is formulated as

(7) $$\mathrm{Levy}(\xi )\sim \frac{u}{|v{|}^{1/2}}$$

(8) $$u\sim N(0,\;\delta_{u}^{2}),\;v\sim N(0,\;\delta_{v}^{2})$$

(9) $$\delta_{u}={\left\{\frac{\Gamma (1+\xi )\cdot sin\left(\frac{\pi \xi }{2}\right)}{\Gamma \left[\frac{1+\xi }{2}\right]\xi \cdot 2^{\frac{\left(\xi -1\right)}{2}}}\right\}}^{1/\xi },\;\delta_{v}=1$$

where Γ(ξ) is the Gamma function and the ξ affects the Levy flight trajectory value.

#### 3.2.4. Artificial Rabbit Optimization Perturbation Strategy

During each iteration of the algorithm, to improve the global optimization capability and convergence speed of the algorithm, this paper uses the mathematical model that simulates the rabbit’s foraging detour in the Artificial Rabbits Optimization [34] (ARO) algorithm to perturb and update the position of individual sparrows in each generation. The formula for correcting the role of individual sparrows in each generation using the artificial rabbit perturbation strategy is given by

(10) $$X_{i,j}^{t+1}=X_{best}^{t}+L\cdot c\cdot (X_{else}^{t}-X_{best}^{t})+S\cdot n_{1}$$

(11) $$L=(e-exp{\left(\frac{t-1}{{iter}_{max}}\right)}^{2})\cdot sin(2\pi r_{2})$$

(12) $$c(k)=\left\{\begin{array}{c} 1,\;\;\;\;k=\mathrm{randperm}(d)\\ 0,\;\;\;\;\;\;\;\;\;\;\;\;\;\;\;\;\;\;\;\;\;\;\;\;\mathrm{else} \end{array}\;\;\;\;k=1,...,d\right.$$

(13) $$S=round(0.5\cdot (0.05+r_{3}))$$

where $X_{else}^{t}$ is the location of the remaining sparrow individuals; $n_{1}$ is a random number that follows a standard normal distribution; L is the step factor; $r_{2}\;and\;r_{3}$ are all random numbers between (0, 1); round denotes rounding up or down; *d* is a variable dimension; randperm(d) is a random integer between 1 and *d* is returned.

**Figure 5:**
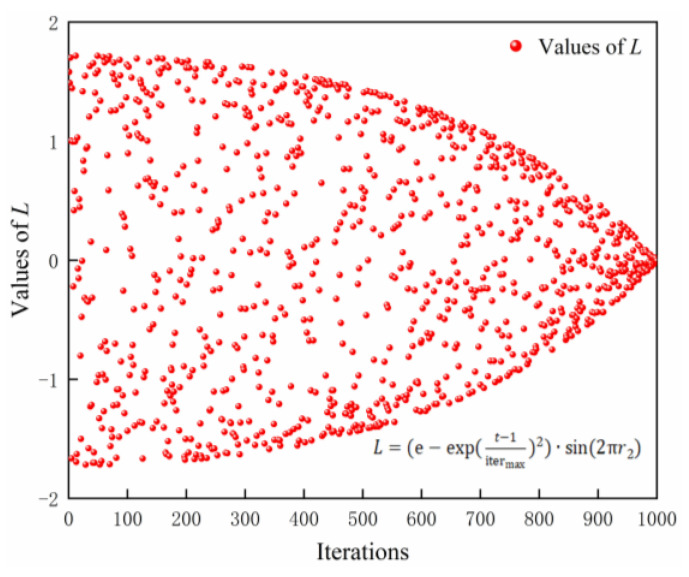
The update method of the step length factor *L* as the number of iterations increases.

Figure 5 shows the variation of the step length factor L with increasing iterations. The improved method of updating the perturbation position of sparrow individuals is carried out from both positive and negative directions, generating a longer step length in the initial iteration and gradually becoming shorter with the increase in the number of iterations. This strategy improves the global search ability of sparrow individuals in the early stage and the convergence speed of the population in the later stage to a greater extent. It can help the ISSA algorithm escape the local optimum for global exploration and local exploitation.

#### 3.2.5. ISSA Performance Evaluation

**Figure 6:**
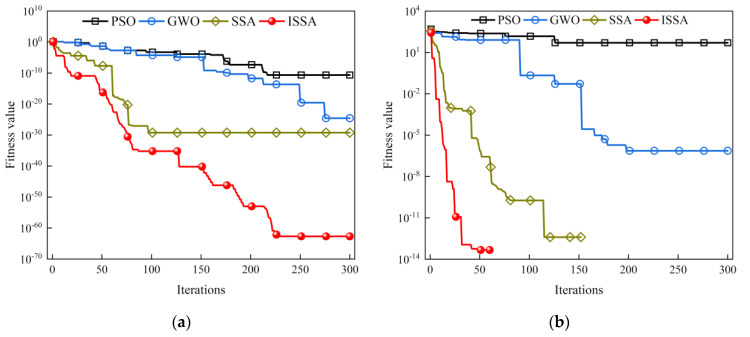
Test unimodal and multimodal function results: (**a**) Schwefel’s Problem function iteration curve; (**b**) Rastigin function iteration curve.

The SSA, particle Swarm Optimization Algorithm (PSO), and Grey Wolf Optimizer (GWO), which evolved by simulating the information exchange and cooperation behavior among biological groups, are all swarm intelligence optimization algorithms with better iterative optimization effects. They are all used in gas monitoring and gas temperature compensation. To verify the iterative optimization performance of the ISSA algorithm, four algorithms, PSO, GWO, SSA, and ISSA, are used for performance evaluation. Schwefel’s Problem single-peak function and Rastrigin multi-peak function are selected among the test functions. The experimental parameters were set as follows: the population size was 21, the number of iterations was 300, and the algorithms were run 30 times, respectively. The test results are shown in Figure 6.

As shown in the test results in Figure 6a, the convergence accuracy and speed are better than the PSO, GWO, and SSA algorithms when solving Schwefel’s problem function with the ISSA algorithm. In Figure 6b, when solving the Rastrigin function, the ISSA algorithm has the fastest convergence speed, which indicates that the ISSA algorithm is more capable of global search and local evolution.

## 4. Model Validation and Discussion

### 4.1. Realization Details

#### 4.1.1. Temperature Compensation Model Prediction Details

**Figure 7:**
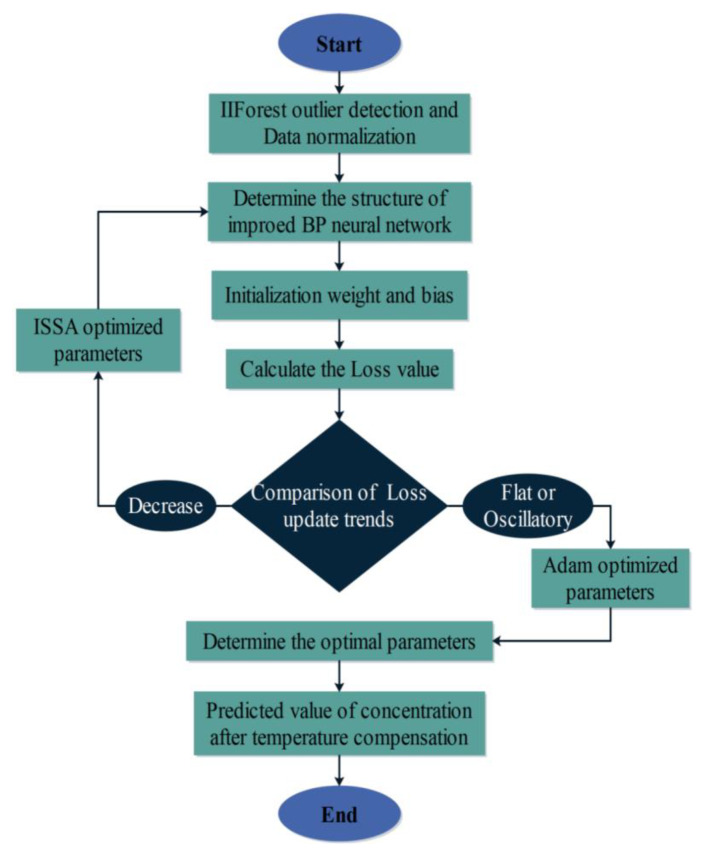
ISSA-BP temperature compensation model prediction flowchart.

In the model study of this paper, we propose to use the non-saturation and smoothness of the Mish function as the activation function of the BP neural network and optimize the BP neural network by combining the ISSA algorithm and the Adam optimizer. As shown in Figure 7 is the flow chart of using ISSA-BP algorithm to establish the temperature compensation model of laser methane sensor, and its specific optimization process is as follows:

Step 1: The 15,810 data detected by the laser methane sensor at different temperatures were divided into training and test samples. The IIForest algorithm was utilized to clean the data of the training samples.

Step 2: The BP neural network hyperparameters are set with the maximum training number and learning rate set to 100 and 0.1, respectively, and the minimum error set to 1 × 10^−5^. We propose using the Mish function as the activation function of the BP neural network and replacing the traditional S-type activation function. The expression for the Mish activation function is

(14) $$f(x)=x\cdot tanh(ln(1+e^{x}))$$

Step 3: Initialize the parameters related to ISSA and initialize the coding work for the weights and thresholds of the BP neural network. The ISSA algorithm sets the population size to be 21 after iterative round-robin trials, the variable dimensions *d* = 21, the maximum number of evolutions to be 150, the population limit pop_max_ = 4, pop_min_ = −4, and 20% of the population to be the explorers, and the rest to be the followers.

Step 4: Initialize the Adam optimizer [35]. The learning rate of the Adam optimizer is set to 0.001, the two moving average coefficients *β*_1_ and *β*_2_ take the values of 0.9 and 0.999, and the smallest actual number of positional stability is 1 × 10^−8^.

Step 5: Calculate the judgment loss function Loss value. When the Loss value shows a decreasing trend, Step 6~Step 12 is performed, and the parameters are updated using ISSA global search. When the Loss value is no longer decreasing, jump to Step 13 and update using Adam optimizer local search.

Step 6: Initializing the population using a quasi-reflective learning strategy.

Step 7: Calculate the fitness of sparrows during foraging and antipredation. Find the location of the best and worst fitness.

Step 8: Sparrow Explorer performs the position update according to Equation (4).

Step 9: The remaining individuals outside of the explorer are followers that follow the explorer for foraging, and their positions are iteratively updated by the formula:

(15) $$X_{i,j}^{t+1}=\left\{\begin{array}{c} Q\cdot \exp\left(\frac{X_{worst}^{t}-X_{i,j}^{t}}{i^{2}}\right),\;\;\;\;\;\;\;\;\;\;\;\;\;\;\;\;\;\;\;\;\;\;\;\;\;\;i>\frac{n}{2}\\ X_{P}^{t+1}+\left|X_{i,j}^{t}-X_{P}^{t+1}\right|\cdot A^{+}\cdot L,\;\;\;\;\;\;\;\;\;\;\;\;\;\;\;\;\;others \end{array}\right.$$

where *X_p_* and *X_worst_* denote the best and worst adaptation searched by the explorer, respectively; A denotes that each element in a 1 × *g* matrix is randomly assigned a value of 1 or −1, and *A*^+^ = *A*^T^(*AA*^T^)^−1^; *L* is a 1 × *d* matrix.

Step 10: The sparrow anti-predator performs position updating according to Equation (6).

Step 11: All individuals were updated with the current optimal position of sparrow individuals using the artificial rabbit perturbation Equations (10)–(13).

Step 12: Fitness update. Determine whether the set maximum number of iterations or the initially set minimum error has been reached. If it is satisfied, then proceed to the next step. Otherwise, return to Step 7.

Step 13: If the value of the loss function Loss varies smoothly, the Adam optimizer is used to search locally for each parameter of the improved BP neural network and update each parameter.

Step 14: The optimal individual fitness was assigned to each parameter of the BP neural network and tested by simulation modeling of the ambient temperature and concentration data, which continued to output the temperature-compensated CH_4_ concentration predictions after the inverse normalization process.

#### 4.1.2. Model Performance Evaluation Index

To evaluate the prediction accuracy of temperature compensation models, the commonly used model evaluation metrics are Mean Absolute Error (MAE), Mean Absolute Percentage Error (MAPE), Root Mean Square Error (RMSE), and Correlation Coefficient (R^2^). The evaluation index formulas are

(16) $$\mathrm{MAE}=\frac{1}{n}\sum_{i=1}^{n}\left|{\hat{y}}_{i}-y_{i}\right|$$

(17) $$MAPE=\frac{1}{n}\sum_{i=1}^{n}\left|\frac{{\hat{y}}_{i}-y_{i}}{y_{i}}\right|\times 100\%$$

(18) $$RMSE=\sqrt{\frac{1}{n}\sum_{i=1}^{n}{\left({\hat{y}}_{i}-y_{i}\right)}^{2}}$$

(19) $$R^{2}=1-\frac{\sum_{i=1}^{n}{\left({\hat{y}}_{i}-y_{i}\right)}^{2}}{\sum_{i=1}^{n}{\left({\bar{y}}_{i}-y_{i}\right)}^{2}}$$

where *y_i_* and $\hat{y}$*_i_* are the actual value of methane concentration and the predicted output, respectively. ${\bar{y}}_{i}$ is the average of the fundamental importance of the experimentally measured CH_4_ and the actual values of gas concentration measured in the test. This paper calculates concentrations in ppm when using MAE, RMSE, and MAPE evaluation indexes.

### 4.2. Comparison Experiment

To verify the effectiveness of the ISSA-BP model in temperature compensation comparatively, we compare and analyze it with existing widely used SVM, Random Forest, BP, and PSO-BP temperature compensation models, respectively. The results of the comparison experiments are shown in Table 4. The table shows that the neural network-based temperature compensation model predictions are distributed on both sides of the standard concentration. The random forest model outperforms the SVM and basic BP neural network models, but the error is larger than the PSO-BP and ISSA-BP models. The predictions of the ISSA-BP temperature compensation model are concentrated near the standard values, and its projections and errors are much better than those of the other models.

The model evaluation results in Table 5 show that the MAE, MAPE, and RMSE of the test samples of SVM, BP, and RF models are much higher than those of the training samples, while the R^2^ is significantly lower than that of the training samples. This indicates that these three models overfit the data during training, resulting in reduced generalization ability and stability and increased error. In contrast, the prediction results of the training and test sets of the ISSA-BP neural network-based temperature compensation model are the same. This indicates that the ISSA-BP model has better learning ability and robustness.

### 4.3. Ablation Experiments

#### 4.3.1. Experimental Results before and after Data Preprocessing

**Figure 8:**
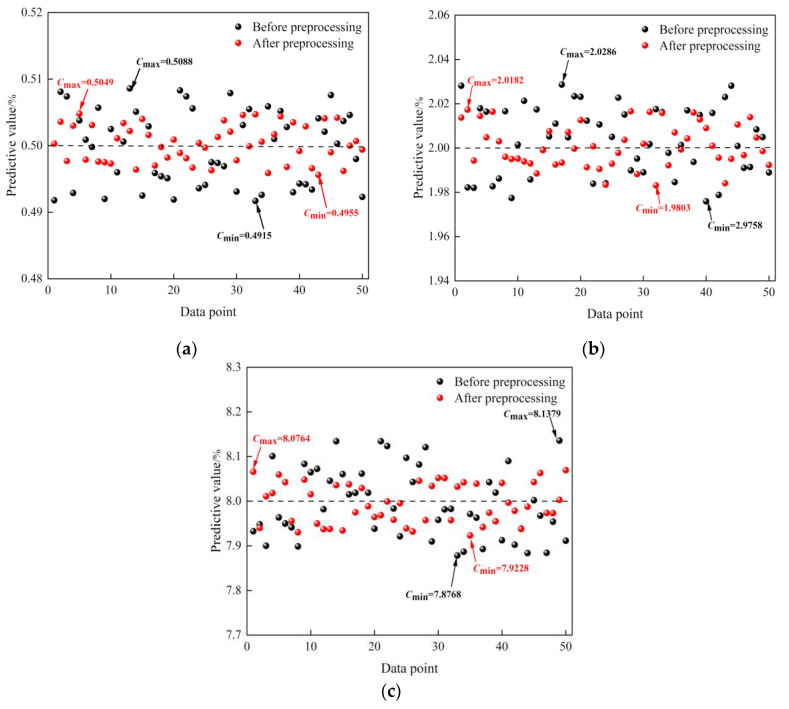
Comparison of model prediction effect before and after data preprocessing: (**a**) prediction results of 0.5% CH_4_ concentration; (**b**) prediction results of 2% CH_4_ concentration; (**c**) prediction results of 8% CH_4_ concentration.

To verify the effect of data preprocessing on the temperature compensation results. The prediction effect of the model before and after data preprocessing is compared and experimentally verified. A comparison of the 50 sets of predicted values near the maximum relative error for each concentration is shown in Figure 8a–c. It can be seen that the prediction of the test set has been improved more after using the IIForest algorithm to remove the outlier data from the training set. Its predicted values are closer to the standard concentrations. The prediction effect of the ISSA-BP model has been significantly improved after data preprocessing. The maximum values expected for each concentration before data preprocessing were 0.5088%, 2.0286%, and 8.1379%, respectively. After data preprocessing, the maximum values predicted by each concentration were 0.5049%, 2.0182%, and 8.0764%, respectively.

The model evaluation index in Table 6 shows that the temperature compensation based on the ISSA-BP Neural Network has better stability and generalization ability. The difference between the evaluation indexes of its prediction effect in the test and training sets is slight. The MAPE values of the training and test sets before and after data preprocessing were reduced by 0.5890% and 0.6160%.

#### 4.3.2. ISSA-BP Ablation Experiments

To further validate the scientific validity of the proposed ISSA-BP temperature compensation model. This section investigates different optimization approaches based on this model, and ablation experiments are carried out on large-scale datasets with different temperatures and concentrations, as follows:

Baseline: Directly use the improved BP neural network to build a temperature compensation model to achieve a laser methane sensor’s predicted output with different temperatures and concentrations.

+Adam optimizer: To solve the BP neural network problem, which has a slow convergence speed and quickly falls into the local optimum, the Adam optimizer is introduced to improve the BP neural network.

+SSA: Due to the Adam-BP temperature compensation model, the Adam optimizer cannot optimize the parameters of the BP neural network well when the value of the loss function Loss is decreasing fast. Therefore, the introduction of SSA is considered for iterative optimization of the weights and thresholds of the BP neural network when the Loss value of the temperature compensation model decreases faster.

Our proposed ISSA-BP Neural Network: To address the shortcomings of the standard SSA algorithm’s global optimization search and local development capabilities. Optimized BP neural networks using our proposed ISSA and Adam algorithms are used to build temperature compensation models, and model validation and evaluation are performed on the established large-scale data.

**Figure 9:**
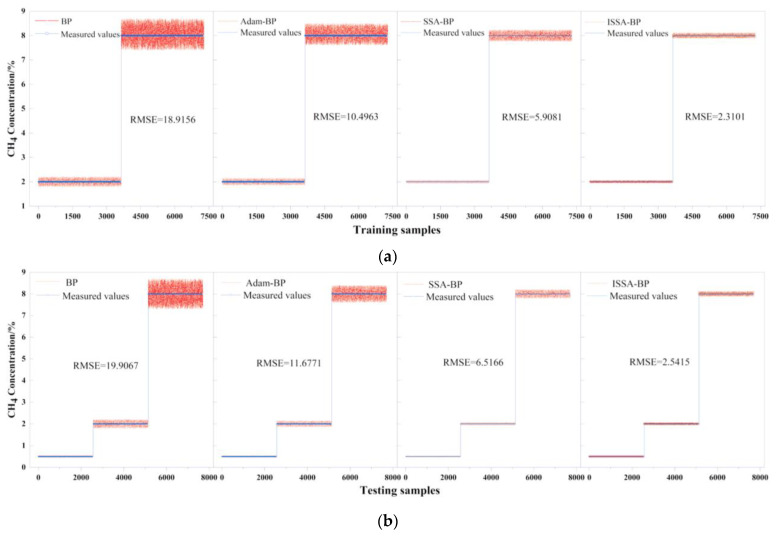
Comparison of corrected results and actual values for four models and the training and testing samples: (**a**) training samples; (**b**) testing samples.

The comparison of the corrected results of each model with the actual values is shown in Figure 9. From the figure, it can be seen that the ISSA-BP temperature compensation model has the best prediction accuracy and stability on the retraining set and the test and training set.

Table 7 lists the highest and lowest predicted values of the three sets of untrained CH_4_ experimental test samples at different temperature intervals of the concentration. As can be seen from the data distribution in the table, there is a slight difference in the prediction effect of the several groups of temperature compensation models performing ablation experiments at room temperature conditions. However, there is a significant gap in the compensation effect for the laser methane sensor’s high- and low-temperature data. With the step-by-step optimization of the model, the prediction effect of the ISSA-BP temperature compensation model reaches the best, and the prediction value fluctuates stably between the standard concentrations.

**Figure 10:**
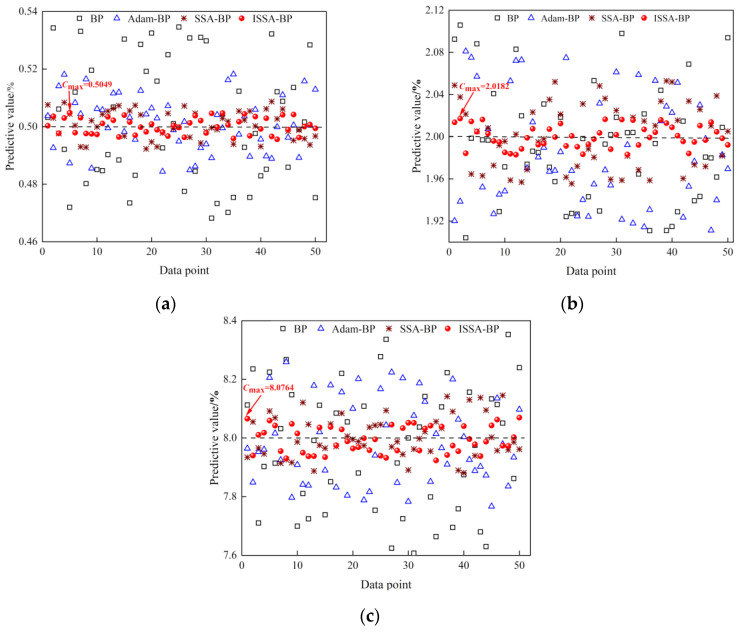
BP, Adam-BP, SSA-BP, and ISSA-BP temperature compensation model prediction output: (**a**) prediction results of 0.5% CH_4_ concentration; (**b**) prediction results of 2% CH_4_ concentration; (**c**) prediction results of 8% CH_4_ concentration.

Figure 10a–c shows the comparison of the results of 50 sets of prediction samples near the maximum relative error after temperature compensation using four temperature compensation models. The ISSA-BP temperature compensation model results are concentrated on both sides of the standard concentration, and the predicted values fluctuate steadily on both sides of the standard value.

**Figure 11:**
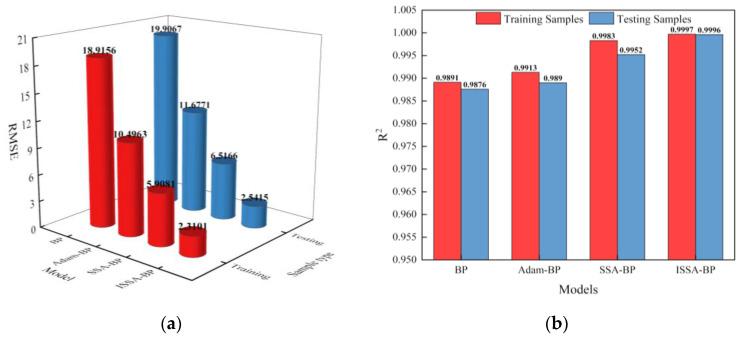
Histogram of RMSE and R^2^ for training and testing the four models: (**a**) Histogram of RMSE for training and testing samples; (**b**) Histogram of R^2^ for training and testing samples.

Figure 11a shows a histogram of the RMSE for the training and testing samples and the four models, while Figure 11b shows a histogram of the correlation coefficient R^2^. As shown in Table 8, to compare the modified performance of each model, the MAE, MAPE, RMSE, and R^2^ of the training and testing samples were calculated for the four models and original data. The MAE, MAPE, and RMSE of the Adam-BP model were more significant than those of the SSA-BP model in both the training and testing phases, and the R^2^ was smaller than the SSA-BP model, indicating that the nonlinear fitting performance of the Adam-BP model was not ideal. In the testing phase, the MAE, MAPE, RMSE, and R^2^ of the SSA-BP model differed less from the training phase, but the error was more significant, and the R^2^ was relatively small.

However, the deviations of MAE, MAPE, and RMSE of the ISSA-BP temperature compensation model in the test phase were all less different from the results of the training phase, and no large overfitting was found. Compared with the original measurement errors, the training samples predicted results with 52.0644 ppm lower MAE, 15.1463% lower MAPE, 61.5973 lower RMSE, and 0.1264 higher R^2^. The test samples indicated effects with 52.0172 ppm lower MAE, 15.5535% lower MAPE, 62.5873 lower RMSE, and 0.1227 higher R^2^. From the overall performance, the ISSA-BP model has a low error, stable model output, high generalization, and high stability. Therefore, the ISSA-BP temperature compensation model has higher prediction accuracy and better equilibrium. The system meets the requirement of compensating for detecting CH_4_ gas concentration under significant temperature variations.

**Figure 12:**
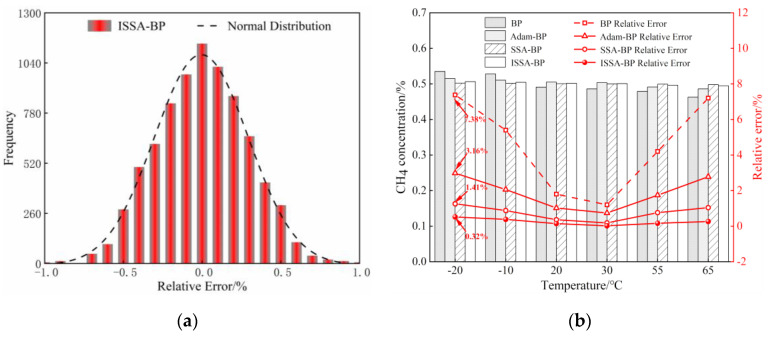
Analysis of data for methane gas temperature compensation: (**a**) Histogram of the prediction error of ISSA-BP model; (**b**) Comparison of model predictions for CH_4_ gas at 0.5%.

Figure 12a shows the histogram of the distribution of relative errors predicted by the ISSA-BP model. Figure 12b compares experimental data and relative errors of the above four models after compensation for 0.5% standard CH_4_ concentration under different temperature conditions. The experimental results show that the relative error between the predicted value and the standard concentration value of the temperature compensation model using ISSA-BP is significantly reduced, and the gas concentration fluctuates within a small range. Based on the above comparison and analysis, the ISSA-BP model is suitable for temperature compensation of CH_4_ gas detection based on TDLAS technology, and the reliability of the system measurement is significantly improved.

### 4.4. Algorithm Utility Analysis

In order to further explore the practical application capability of the temperature compensation model algorithm proposed in the article, we chose the BP neural network, which is suitable for embedded hardware implementation, as the basis and analyzed in detail the performance of the ISSA-BP neural network model in practical applications. Considering the importance of the number of parameters, operational complexity, and inference speed of the established temperature compensation model for practical applications, we conducted the following comprehensive analysis as follows:

1. Number of operational parameters. According to the analysis in Section 3.1, the ISSA-BP neural network temperature compensation model contains two input layer neurons, five hidden layer neurons, and one output layer neuron. Every two connected neurons have operational parameters for weights, and neurons in the remote and output layers contain operational threshold parameters. A smaller number of parameters means lower model complexity and faster training speed, which helps reduce the risk of model overfitting and facilitates deployment in environments with limited hardware computing resources.
2. Number of model operations. In the neural network model structure, each connected neuron node performs a multiplication operation with the neural network weights and an addition operation with the threshold value. Therefore, our proposed temperature compensation model requires 15 multiplication operations, six addition operations, and six operations of the activation function during forward propagation. This indicates that the model can enhance its nonlinear fitting ability by activating the function in the operation and showing high computational efficiency, which is suitable for scenarios requiring fast response.
3. Inference speed and practical application. The hardware temperature compensation based on the ISSA-BP model structure takes only about 40 milliseconds to compute the prediction process on an MCU chip running at 8 MHz. This short prediction inference time is suitable for real-time application environments, and different hardware devices will also exhibit different inference speeds.
4. Hardware compatibility. The model’s simplicity implies lower hardware requirements, making it easier to deploy on various devices, including in environments such as embedded systems.

As shown in Table 9, the above analysis concludes that the temperature compensation model based on the ISSA-BP neural network architecture has good practical advantages regarding the number of parameters, computational efficiency, hardware compatibility, and so on. The model is suitable for application scenarios requiring fast response, such as real-time temperature compensation or pressure compensation of laser methane sensors.

## 5. Discussion and Conclusions

This paper proposes a temperature compensation method based on the ISSA-BP Neural Network model and a large-scale measured high- and low-temperature methane gas dataset. The prediction accuracy of the temperature compensation of the laser methane sensor under a wide range of temperature application conditions is improved. Firstly, the improved isolation forest algorithm is used to eliminate the outliers in the laser methane sensor data training set under high- and low-temperature conditions to reduce the influence of data noise on the training effect of the temperature compensation model. Secondly, the BP neural network is improved regarding the weight updating method. The original S-type activation function is replaced with the Mish activation function, and the improved BP neural network is optimized using the ISSA and Adam algorithms. The prediction performance and generalization ability of temperature compensation are greatly improved. Finally, the model’s applicability in the field of temperature compensation of laser methane sensors is verified by the established 15,810 sets of experimental data. The experimental results show that the linear regression coefficients of the temperature compensation model selected based on IIForest outlier detection and ISSA-BP neural network for the training and test sets reach 0.9997 and 0.9996, respectively. Compared with other temperature compensation models, the proposed method achieves higher prediction accuracy and more vital generalization ability, illustrating its effectiveness.

## Figures and Tables

**Table 1:** Differentiation of training and test data samples.

Datasets	Temperature	Concentration/%	Samples
Training samples	−20~−10 °C	Measured values of 2% and 8.0% CH_4_ concentration	2800
	15~30 °C		2800
	55~65 °C		2800
Test samples	−20~0 °C	Measured values of 0.5%, 2.0% and 8.0% CH_4_ concentration	2470
	10~30 °C		2470
	40~65 °C		2470

**Table 2:** Data distribution of the training set after outlier removal.

Datasets	Temperature	Concentration/%	Samples
Training samples	−20~−10 °C	Measured values of 2% and 8.0% CH_4_ concentration	2653
	15~30 °C		2694
	55~65 °C		2660

**Table 3:** Cyclic test results of different hidden layer nodes.

Nodes	3	4	5	6	7
MSE	2.95 × 10^−3^	2.32 × 10^−4^	3.23 × 10^−5^	9.61 × 10^−5^	4.33 × 10^−4^

**Table 4:** CH_4_ concentration output predicted value after temperature compensation.

CH_4_ Concentration	Algorithm	Predicted Value of CH_4_ Concentration/%		
		−20~0 °C	10~30 °C	40~65 °C
0.5%	SVM	0.5178~0.5465	0.4911~0.5089	0.4568~0.5141
	BP	0.5147~0.5411	0.4863~0.5081	0.4632~0.5125
	Random Forest	0.5102~0.5251	0.4931~0.5076	0.4687~0.5098
	PSO-BP	0.5067~0.5113	0.4972~0.5041	0.4887~0.5052
	ISSA-BP	0.4991~0.5049	0.4996~0.5034	0.4955~0.5025
2.0%	SVM	2.0623~2.1601	1.9963~2.0110	1.8633~2.0953
	BP	2.0798~2.1493	1.9981~2.0094	1.8895~2.0866
	Random Forest	2.0312~2.1022	1.9961~2.0112	1.9411~2.0791
	PSO-BP	2.0192~2.0511	1.9933~2.0098	1.9883~2.0252
	ISSA-BP	1.9921~2.0182	1.9992~2.0105	1.9803~2.0098
8.0%	SVM	8.1088~8.5262	7.9813~8.0166	7.5351~8.1211
	BP	8.0994~8.4983	7.9877~8.0160	7.6043~8.1088
	Random Forest	8.0692~8.3688	7.9828~8.0158	7.7102~8.0868
	PSO-BP	8.0594~8.2003	7.9891~8.0136	7.8866~8.0534
	ISSA-BP	7.9893~8.0764	7.9916~8.0123	7.9228~8.0398

**Table 5:** Performance evaluation index of five temperature compensation models.

Model	MAE (ppm)		MAPE (%)		RMSE (ppm)		R^2^ (%)	
	Training	Testing	Training	Testing	Training	Testing	Training	Testing
SVM	15.3743	22.9878	3.4584	7.0947	19.0161	28.2363	0.9789	0.9669
BP	15.1560	21.6888	3.2768	6.6253	18.6892	24.1691	0.9841	0.9722
Random Forest	13.7743	17.1658	2.9276	5.4241	15.6816	19.2636	0.9875	0.9788
PSO-BP	6.7234	9.0197	2.4041	4.4661	9.4515	11.3161	0.9891	0.9872
ISSA-BP	1.2813	1.4525	0.2721	0.2961	2.3101	2.5415	0.9997	0.9996

**Table 6:** Indicators for model evaluation before and after data preprocessing.

Experiment	MAE (ppm)		MAPE (%)		RMSE		R^2^ (%)	
	Training	Testing	Training	Testing	Training	Testing	Training	Testing
Before preprocessing	2.6793	2.7655	0.8611	0.9121	3.9123	4.1211	0.9995	0.9993
After preprocessing	1.2813	1.4525	0.2721	0.2961	2.3101	2.5415	0.9997	0.9996

**Table 7:** Predicted output of CH_4_ concentration for ablation experiments.

CH_4_ Concentration	Algorithm	Predicted Value of CH_4_ Concentration/%		
		−20~0 °C	10~30 °C	40~65 °C
0.5%	BP	0.5138~0.5356	0.4913~0.5077	0.4682~0.4965
	Adam-BP	0.5061~0.5198	0.4975~0.5052	0.4839~0.5093
	SSA-BP	0.5022~0.5093	0.4991~0.5058	0.4916~0.4985
	ISSA-BP	0.4991~0.5049	0.4996~0.5031	0.4955~0.5025
2.0%	BP	2.0541~2.1215	1.9896~2.0104	1.9033~2.0621
	Adam-BP	1.9872~2.0813	1.9965~2.0096	1.9104~2.0224
	SSA-BP	1.9951~2.0563	1.9988~2.0084	1.9462~2.0156
	ISSA-BP	1.9921~2.0182	1.9992~2.0081	1.9803~2.0098
8.0%	BP	8.0681~8.4056	7.9877~8.0143	7.6043~8.0988
	Adam-BP	8.0264~8.2603	7.9869~8.0126	7.7565~8.0401
	SSA-BP	8.0212~8.1452	7.9901~8.0124	7.8688~8.0416
	ISSA-BP	7.9893~8.0764	7.9916~8.0123	7.9228~8.0398

**Table 8:** Performance evaluation index of four temperature compensation models.

Model	MAE (ppm)		MAPE (%)		RMSE		R^2^ (%)	
	Training	Testing	Training	Testing	Training	Testing	Training	Testing
Original	53.3457	53.4697	15.4184	15.8496	63.9074	65.1288	0.8733	0.8769
BP	10.0317	13.7018	3.8455	4.7069	18.9156	19.9067	0.9891	0.9876
Adam-BP	6.3808	7.7659	2.4442	3.5784	10.4963	11.6771	0.9913	0.9890
SSA-BP	3.3656	3.538	1.1638	1.2093	5.9081	6.5166	0.9983	0.9952
ISSA-BP	1.2813	1.4525	0.2721	0.2961	2.3101	2.5415	0.9997	0.9996

**Table 9:** Analysis of parameter operations based on ISSA-BP neural network model architecture.

Practicality Analysis	Descriptions	Value
Number of parameters	Total number of weights and bias values in the model	21
Additive and multiplicative operations	Total number of multiplication and addition operations during forward propagation	21
Activation function operation	Number of operations using the activation function	6
Forecasted time	Time taken to complete one temperature compensation prediction	40 ms

## Data Availability

Data are contained within the article.
